# Observational study on the efficiency of Neonatal Emergency Transport in reducing mortality and morbidity indexes in Sicily

**DOI:** 10.1038/s41598-021-99477-5

**Published:** 2021-10-12

**Authors:** Raffaele Falsaperla, Giovanna Vitaliti, Barbara Amato, Marco Andrea Nicola Saporito, Laura Mauceri, Federica Sullo, Milena Motta, Bruna Scalia, Federica Puglisi, Martina Caccamo, Maria Grazia Longo, Valentina Giacchi, Carla Cimino, Martino Ruggieri

**Affiliations:** 1 Neonatal Intensive Care Unit and Neonatology, San Marco Hospital, Azienda Policlinico “Rodolico-San Marco”, Catania, Italy; 2 Pediatrics Unit and Pediatric Emergency Room, San Marco Hospital, Azienda Policlinico “Rodolico-San Marco”, Catania, Italy; 3 Unit of Rare Diseases of the Nervous System in Childhood, Department of Clinical and Experimental Medicine, Section of Pediatrics and Child [AQ03] Neuropsychiatry, University of Catania, Catania, Italy

**Keywords:** Neonatology, Neonatal brain damage

## Abstract

In these last 25 years, the Neonatal Emergency Transport (NET) service has been widely improved in Italy. To date, all National areas are covered by a NET service; 53 NET centers have been activated in all the Italian territory. Herein, the authors present an observational study to evaluate the rate of infantile mortality after introduction of NET in Sicily, and to study the efficiency of this service in reducing these rates of mortality in vulnerable neonates, transported from primary care birth centers to tertiary facilities to undergo to specialized NICU assistance. All neonates who required an emergency transport by NETS were included. No exclusions criteria were applied. Demographic and regional infantile mortality data, expressed as infant mortality rate, were selected by the official government database (ISTAT- National Statistic Institute—http://www.istat.it). All data were respectively divided into three groups: data concerning transport, clinical condition, and mortality of the transported patients. We transported by NET 325 neonates. The analysis of the infant mortality rate (per 1.000 live births) in Catania from 2016 to 2018 was reduced compared to the same rate calculated before NETS activation (4.41 index before 2016 vs 4.17 index after 2016). These data showed an increase in other provinces (Enna, Caltanissetta, and Agrigento). 61% of neonates showed a respiratory disease. During the study period the proportion of neonates with a Mortality Index for Neonatal Transportation—MINT < 6 has been reduced, while there was an increase of neonates with higher Transport Risk Index of Physiologic Stability-TRIPS score results. The slight decrease of infantile mortality in Catania during the first three years after introduction of NET follows the same trend of all Italian territories, showing the importance of this service in reducing infantile mortality.

## Introduction

In December 2010, an agreement among the Italian Government, Regions, Cities and Provinces was signed. This document described the "Guidelines to promote and improve quality, safety, and pertinence of health interventions at birth, and to promote a decrease of caesarean sections". The aim of this agreement was to reorganize the regional network of birth centres, including the introduction of a "hub and spoke model" and the identification of those centres that would have been involved in the Neonatal Emergency Transport Network.

In these last 25 years, the Neonatal Emergency transport (NET) service has been widely improved in Italy. To date, all National areas are covered by a NET service; 53 NET centres have been activated in all Italian territories. The NET centre of Catania was one of the last to be activated in 2016. Its activation urged after the death of a term newborn during ambulance emergency transport (The Nicole Case). The child was born in a private birth centre (I level facility), and she was transferred by ambulance to a nearby city as there were not NICU beds available in Catania and its surrounding areas. Unfortunately, the infant died in ambulance during the transport (Quotidiano “La Repupplica” sezione cronaca 12/02/2015).

A Neonatal Emergency Transport represents a system of transport dedicated to those neonatal acute diseases and emergency situations that are not predictable before birth^1–4^.

The transport of critically ill neonates from initial stabilizing hospitals to specialized neonatal facilities is fundamental to guarantee neonatal on-going and specialized medical cares and surgical management, above all if initial hospitals are not provided with tertiary care facilities.

Neonatal Emergency Transport reduces neonatal morbidity and mortality of critically ill neonates born in primary care birth centres and increases accessibility of health facilities by effective referral linkages through efficient transport systems. This network is usually covered by effective linkages of community-based models, facility based-models, primary to tertiary care facilities, and public sector emergency services^5–7^.

In this context, transport distances can be extensive and require transition between different modes of transportation, with exposure to environmental factors, in combination with a potential worsening of the underlying disease, these neonates may be at risk of experiencing adverse events up to death during transport^8–12^.

Nevertheless, to our knowledge there is no recent literature about this topic and new data are mandatory to establish a concern in regards.

Herein, the authors present an observational study to evaluate the rate of infantile mortality after introduction of NET in Sicily, and to study the efficiency of this service in reducing the rate of mortality in vulnerable neonates, transported from primary care birth centers to tertiary facilities to undergo specialized NICU assistance.

## Materials and methods

The present study is a retrospective analysis of all Neonatal Emergency Transports (NETs) performed by our Neonatal Emergency Transport Service (NETS), in the NICU of San Marco Hospital, Catania, Italy, from January 2016 to December 2020.

All neonates who required an emergency transport by NETS, according to pre-determined NET activation clinical criteria^13^, were included. The criteria for emergency transport were: “1. presence of respiratory distress; 2. weight under 1500 gr; 3. weight between 1500 and 2000 gr with mild pathology; 4. prematurity with a GA ≤ 34 weeks; 5. asphyxia with need for requiring advanced resuscitation; 6. congenital cyanogen or duct-dependent heart disease; 7. surgical conditions that could compromise vital functions; 8. complex malformations that could compromise vital functions; 9. intubated patients and/or children with central infusion lines; 10. compromised vital signs.

No exclusions criteria were applied.

Demographic data and Regional infantile mortality data, expressed as infant mortality rate (Number of infant deaths per 1000 registered live births.) were selected by the official government database (ISTAT- National Statistic Institute—http://www.istat.it).

We analyzed data of infantile mortality recorded in the ISTAT database (per 1000 alive births) in the cities of Catania, Enna, Caltanissetta, and Agrigento. These data were updated to 2018.

Data were collected by analyses of transport records and clinical folders of each patient. They were recorded in schedules included in the clinical folders of each patient. Data were then collected in an excel database in the personal computer of the corresponding author.

For each patien we collected the following information: level and site of the initial stabilizing birth centre, and those of tertiary care facilities after transport; transport interval time; clinical conditions of transported neonates (gestational age, birth weight, diagnosis at birth, age at transport, presence of respiratory, surgical, neurologic diseases, and/or cardiac abnormalities); specific indexes to evaluate the infant clinical condition before transport (MINT score: Mortality Index for Neonatal Transport score), and during transport (TRIPS score: Transport Risk Index of Physiologic Stability score); neonatal mortality and morbidity outcomes (death or days of hospitalization in NICU).

Data on MINT and TRIP scores were collected according to international standardized prefilled schedules^14,15^ and then were added to the excel database in the personal computer of the corresponding author.

The Emergency Operative Unit, identified by the 118 national unified call number, was the coordinator of all the Regional NETs, providing 24 h service.

The NET team for each transport included: a NICU neonatologist, a NICU nurse, a 118 assistant, and a 118 driver. The neonatologist and the NICU nurse had undergone training specifically in a transport environment. In addition, apart from the ambulance services, the transport service utilized an helicopter in case of extremely emergent clinical conditions.

A properly equipped ambulance was identified as the mean of transport, in which a single neonatal incubator could be carried.

The present study was approved by the Ethical Committee of the University of Catania, Italy. All research was performed in accordance with relevant guidelines/regulations: Informed consent was obtained from all the caregivers of the studied patients. The research was performed in accordance with the Declaration of Helsinki guidelines.

## Statistical analysis

Statistical analysis was performed using Microsoft Excel 2011 for Mac.

MINT score was collected at the first telephone contact by the referring hospital with the transport team^14^.

TRIPS score was used to predict seven-day mortality and to assess the clinical severity of infants at the time transport was performed. At the end of transport, it was re-evaluated for transport effectiveness^15^.

Descriptive statistics were calculated for all clinical variables. Qualitative variables are represented as a percentage and quantitative variables as mean ± standard deviation. Kolmogorov–Smirnov One-Sample Test and statistics were used for testing normal distribution of quantitative variables.

### Ethics approval and consent to participate

The study was approved by the Ethical Committee of the University of Catania, Italy. All parents of the included patients gave their consent to participate to the present study.

### Consent for publication

All parents of the included patients gave their consent for publication of the present data in anonymous form.

## Results

### Demographic characteristic

In our study, we included 325 neonates, who required NET from primary care birth centres to tertiary health facilities, for acute onset of non-predictable diseases.

**Figure 1:**
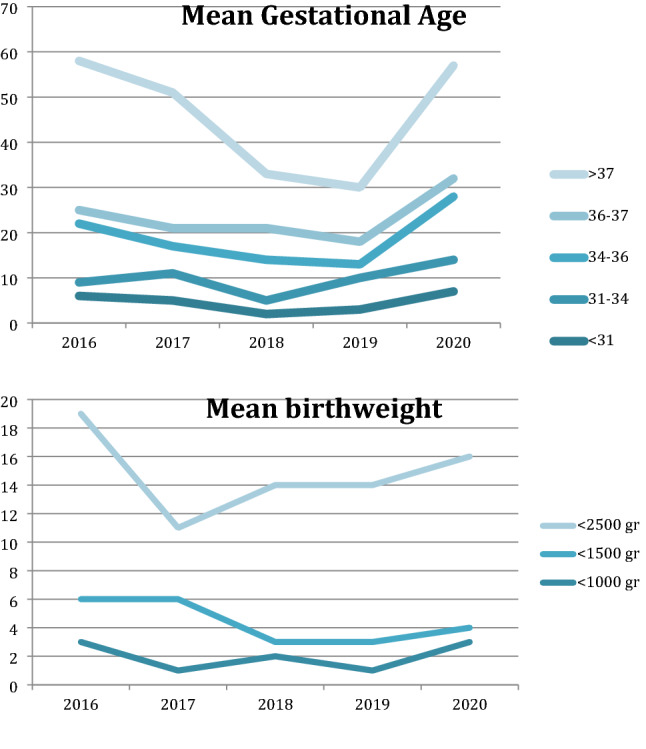
overview in demographic data from 2016 to 2020 of patients requiring NETS included in our study. (**a**) shows overview the trend of the mean GA, and (**b**) mean birth-weight during the study period. The average birth weight of infants is 2484 g (600–5050 g). The proportion of infants with birthweight < 1000 gr is stable at 4.5%; the proportion of infants weighing < 1500 gr was 4.5% in 2016 and 1.6 in 2020%; the proportion of infants weighing < 2500 gr is stable 18–19%.

Mean gestational age was 35 ± 4 weeks of gestation at birth. The proportion of neonates with a gestational age (GA) lower than 31 weeks was 10.6% in 2016, and 9.3% in 2009 respectively; patients with a GA between 31 and 34 weeks were 20–21% per year; patients with a GA between 35 and 37 weeks were 6.6% in 2016, and 8.8% in 2020 respectively; patients with a GA over 37 weeks increased from 37.8% up to 51.5% from 2016 to 2020 (Table 1, Fig. 1).

**Table 1:** Gestational age (GA) (a), birthweight (b) of patients transferred during 2016–2020.

Gestational age (weeks)	2020	2019	2018	2017	2016
Mean ± SD	35 ± 4	36 ± 5	36 ± 3	35 ± 5	35 ± 3
< 31	7	3	2	5	6
31–34	7	7	3	6	3
34–36	14	3	9	6	13
36–37	4	5	7	4	3
> 37	25	12	12	30	33

Birth weight (g)	2020	2019	2018	2017	2016
Mean ± SD	2667.5 ± 921	2456 ± 866	2567 ± 839 gr	2388 ± 851	2345 ± 825
< 1000 gr	3	1	2	1	3
< 1500 gr	1	2	1	5	3
< 2500 gr	12	11	11	5	13

The mean birth weight of the included neonates was 2.484 ± 850 gr (with a range between 600 and 5050 gr). Among included neonates, 4.5% had a birth weight lower than 1000 gr; 4.5% in 2016 and 1.6% in 2020 respectively had a birth weight lower than 1500 gr; 18–19% had a birth weight lower than 2500 gr during the whole study interval (Table 1, Fig. 1).

### Mortality before and after NET opening implementation

The analysis of the infant mortality rate (per 1.000 alive live births) in Catania from 2016 to 2018 was reduced compared to the same rate calculated before NETS activation (4.41 index before 2016 vs 4.17 index after 2016). The same analysis was carried out also in Enna, Caltanissetta and Agrigento in which NETS is not yet active. These data showed an increase in infant mortality rate from 2.63 to 3.67 in Enna, from 4.22 to 4.77 in Caltanissetta and from 4.14 to 4.5 in Agrigento, respectively (Table 2).

**Table 2:** The ISTAT data related to the infant mortality rate (per 1.000 live births) of the province of Catania, Enna, Caltanissetta and Agrigento available until 2018.

Neonatal mortality rate (per 1.000 birth)	2013	2014	2015	2016	2017	2018
Catania	4.43	4.8	4.3	4.52	3.72	4.28
Enna	3.18	3.95	3.78	4.15	4.82	6.04
Caltanissetta	4.81	5.16	2.63	6.8	3.54	3.97
Agrigento	4.73	2.18	5.53	5.07	3.85	4.6

In our NET centre, 9% of neonates died between 2019 and 2020 (n = 4 in 2019, and n = 2 in 2020).

### Analysis of morbidity

38% of neonates was dismissed from our Neonatal Intensive Care Unit within 10 days of hospitalization; 43% was hospitalized for a period between 10 and 20 days; 10% was hospitalized for 20–40 days; 14% had an interval of hospitalization of more than 40 days (Table 3).

**Table 3:** Outcome of neonatal patients admitted to tertiary health facilities after NET.

Outcome	2019	2020
Number of dead neonates	4	2
Interval of hospitalization < 10 days	15	10
Interval of hospitalization between 10 and 20 days	4	14
Interval of hospitalization between 20 and 40 days	3	4
Interval of hospitalization > 40 days	4	5

Neonatal diseases requiring NET activation included: respiratory diseases secondary to prematurity in 65% and 57% of cases, in 2016 and in 2020 respectively; surgical diseases in 7% and 11% of cases, in 2016 and in 2020 respectively, with a similar proportion for neurologic diseases; cardiac problems in 7% and 11% of cases, in 2016 and in 2020 respectively.

### Analysis of data of transport safety and associated risks

During the study period, the mean percentage of neonates transported per year was 0.9%, and this data was consistent over the full study period. Among all transports, 59% and 48% were activated in 2016 and 2020 respectively. All patients (100%) were transferred to a tertiary care neonatal centre where a Neonatal Intensive Care Unit was present and no back-transports to primary care birth centres were recorded.

In our study the NET service covered 12 Spoke centres that were distant from the Hub one from 6 to 78 kms. The city of Ragusa was included in this area, but our NET service could not easily reach those birth centres as the time of transport exceeded 60 min of interval. Among the included Spoke centres, 4 had a number of births less than 500 per year.

The mean Interval time to reach the birth centre was about 70 min (62 ± 32 min and SD in 2016; 78 ± 34 min and SD in 2020); the mean Interval time of transport to the tertiary care centre was about 30 min.

The mena time to resuscitate the patient at arrival to the birth center was 45 min.

The mean of transport for all patients was ambulance car. No patient required transport by helicopter.

**Figure 2:**
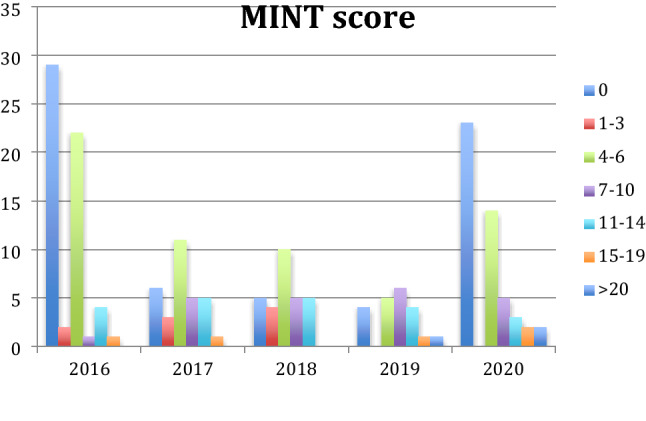
Overview of MINT score during the study period. During the study period the percentage of newborns with null MINT decreased from 44 to 36%; the proportion of infants with MINT between 4 and 6 decreased from 33 to 22%; the percentage of infants with MINT between 7 and 10 increased from 1.5% to 7.8%; infants with MINT greater than 15 increased from 1.5 to 6.2%.

In our study, we used the "Mortality Index for Neonatal Transport" score (MINT score) to study the severity of the neonatal disease requiring NET at birth. We found that the proportion of null MINT had a decrease during the study period, from 44% in 2016 to 36% in 2020; the proportion of MINT between 4 and 6 points was reduced from 33 to 22% in the same period; MINT with a score between 7 and 10 had an increased proportion from 1.5 to 7.8% during the same period; neonates with MINT over 15 points were increased from 1.5% to 6.2% (Fig. 2). The analyses performed showed consistent trends over the full study period (Fig. 2).

**Figure 3:**
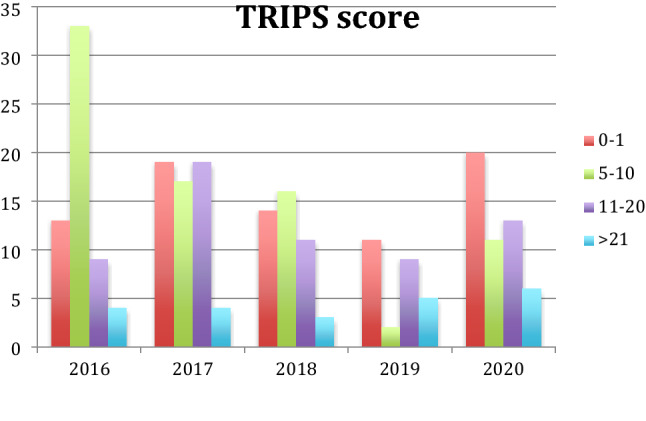
Changes in TRIPS score during the study period. Data regarding the TRIPS score during the study period, show an increase of infants with TRIPS 0–1 from 19 to 31%, the proportion of infants with TRIPS 5–10 reduced from 50 to 17%, the proportion of infants with TRIPS 11–20 increased from 13 to 20%, as well as the proportion of infants with TRIPS > 21 from 6 to 9.3%.

As far as data on "Transport Risk Index of Physiologic Stability" score were concerned (TRIPS score), during the study period we found an increased percentage of neonates with TRIPS 0–1 from 19% up to 31%; the proportion of neonates with TRIPS score between 5 and 10 showed a decrease from 50 to 17%; neonates with TRIPS between 11 and 20 increased from 13 to 20%; patients with TRIPS more than 20 increased from 6 to 9.3% (Fig. 3). The analyses performed showed consistent trends over the full study period (Fig. 3).

## Discussion

We described our experience as NET centre in Catania during an interval time of 5 years. In our centre, we have performed about 65 transports per year. Our NET centre is included among 18/53 Italian centres with a medium number of transports between 50 and 100 per year.

In our study, the number of transports from a I level birth-centre to a tertiary care facility has decreased of about 10% from 2016 to 2020; contemporary we found that the medium interval time of transport has increased from 62 ± 32 min ± SD to 78 ± 34 min ± SD. This was probably due to an increased number of calls from more distant Spoke centres and a reduction of calls from Private Birth-Centre inside the metropolitan area.

In a recent study, performed in California, USA, authors evaluated clinical risks for neonates during emergency transports, and they have not found any increase of clinical risks according to the interval time of transport^16^. A worst clinical outcome seems instead to be associated with neonatal groups according to the severity of their disease at birth^16^. This difference might be explained by two factors: in the USA study, patients could be transferred within 7 days after birth, and therefore well-stabilized and treated with a proper first assistance in the birth-centre, while in our study all patients were transferred within 10 h after birth, in a more vulnerable époque; secondly in the USA study, all transports over 60 min were associated with clinical deterioration, therefore a lower number of long-way transports were performed with respect to our study.

The description of risk factors associated with a clinical decline during a critical care transport is mandatory to guarantee a better management of these neonates. To identify these factors, studies on high-risk neonatal populations, including preterm neonates and/or very low birth weight neonates, should be performed.

### Analysis of mortality before and after NET activation in Sicily

In recent years, numerous NETS centres have been progressively activated in Italy, according to each regional legislature.

In Sicily, in 2015, a female neonatal child died after birth during an emergency transport in ambulance. The child was born on term, with asphyxia at birth, in a I level birth private clinic, in the centre of Catania. The patient needed resuscitation and admission to a nearby NICU. For the lack of availability of NICU beds in Catania and its surroundings, the child was then transferred to Ragusa (with a transfer interval time higher than one hour); yet during the transport died. This case is known on a National scale as "the Nicole case", and it was the trigger to speed up the introduction of NETS centres in Sicily.

In regards, after the death of Nicole, the Italian National Ministry of Health distrust the Sicilian Region not to have improved its birth assistance network, according to 6 urgent indexes introduced in the Italian legislature on the 30th of June 2015.

Sicily therefore had to reorganize the system of maternal and neonatal emergency transports, according to the abovementioned legislature, which details were published in the Official Journal of the Sicilian Region, number 22, on 29 May 2015. This legislature reported the activation of 5 STEN centres, among which Catania had to cover the Oriental Sicily area.

The results of our study showed that in Catania the mean index of infantile mortality had a decrease from 2016 to 2018 (4.17 infant deaths per 1000 neonatal birth), while from 2013 to 2015 the same index was 4.41. In other cities (Enna, Caltanissetta, and Agrigento), during the same period we observed an increase of neonatal mortality from 2.63 infant deaths per 1000 neonatal births up to 3.67 in Enna, from 4.22 to 4.77 in Caltanissetta, and from 4.14 to 4.5 in Agrigento (Table 2).

The slight reduction of infantile mortality in Catania during the first three years after introduction of NETS is in accordance with trends in the rest of Italy: this trend was reversely evidenced in Enna, Caltanissetta, and Agrigento, where NETS was not activated.

In our study we evidenced that the decrease of the infantile mortality was significant in those area where NETS centres were activated, providing then critical care transports in shorter time intervals. In those cities distant from a NETS centre, requiring longer transport time intervals, the infantile mortality index was increased.

These results evidence the need to improve the neonatal transport network and to reduce the transport timing, in order to reach a time interval under 60 min. Nevertheless, these data are only preliminary, and more studies are mandatory to confirm the efficiency of NETS in reducing neonatal mortality.

Mortality in the first month of life after birth represents the major component of infantile mortality, and it seems strictly related to the quality of assistance during pregnancy and at delivery. According to a Pilot Study on Perinatal Mortality Surveillance (SPItOSS), performed in Lombardy, Tuscany and Sicily between 2017 and 2019, financed by the Italian Ministry of Health and coordinated by the Italian Superior Health Institute, perinatal deaths in Italy show a higher incidence than maternal ones^17^.

In Italy we record a number of perinatal deaths of about 1800 neonates per year, while maternal deaths are 40/year.

In 2011, the ISTAT introduced estimates of perinatal mortality according a monthly analysis of demographic events and analysis of death causes. The rate of perinatal deaths in Sicily between 2000 and 2013 was higher than in other Italian regions^17^. These differences reflect the heterogeneity of the quality of perinatal health assistance in different Italian territories, rising concerns about the efficiency and development of an avant-garde health network in the South of Italy, including hospitals with low numbers (< 500) of deliveries per year^18^.

In the SPItOSS study, Tuscany was the region with the highest number of birth centres performing more than 1000 deliveries per year (56.5% of all Italian birth centres), followed by Lombardy (46.5%), while Sicily had the lowest percentage (35.7%). Nevertheless, birth centres with a number of deliveries under 500 are still numerous (17.4% in Tuscany, 11.9% in Sicily, and 6.9% in Lombardy). These proportions are still high according to the Italian Committee for Perinatal Assistance (Comitato Percorso Nascita nazionale (CPNn)), which foresees the presence of these structures only in areas sited in geographic locations difficult to reach (artt. 1 DM 11/11/2015).

Our country still lacks of systems of surveillance of perinatal mortality, according to confidential analyses of perinatal deaths. Nevertheless, the quality of this kind of surveillance is important to provide important information on perinatal assistance, to improve its efficiency and safety, and prevent predictable perinatal deaths^19^.

In Italy, only in Emilia-Romagna, in 2008, a system of surveillance of perinatal mortality was introduced, aiming to spread the consciousness of this health problem, and to improve the diagnostic and therapeutic follow-up. In 2020, the same region included in this surveillance also neonatal deaths within the first week of life, according to the guidelines promoted by the SPItOSS study^17^.

### Analysis of morbidity and safety of transport

In our study we found an increase of transports of term babies. Respiratory diseases associated with preterm birth were the main health problems requiring a neonatal emergency transport. Among respiratory diseases, respiratory distress syndrome, meconium aspiration, pneumonia, and pneumothorax were the most common.

Literature data confirm our findings; in regards, Henry e Trotman in 2017 found that 60% of neonatal transports were activated for respiratory problems^20^.

In 2019 Leung et al. analyzed data on neonatal transports from I level birth-centres to the NICU of the Queen Mary Hospital of Hong Kong^21^. They found that the most common diseases requiring NET were: cardiac diseases (45.7%), surgical interventions (28.1%), and respiratory diseases (16.0%)^21^.

In 2018, Frid et al. published different results, showing that extreme prematurity (22%), neonatal asphyxia requiring hypothermia (22%), and respiratory distress (16.0%) were the most common problems for which NETs were performed^22^.

Kempley et al., in 2004, published a similar study in UK, showing that most urgent transfers were for the baby to receive neonatal surgery (41%) or neonatal intensive care (35%), but other important reasons included cardiac or neurological problems or the need for extracorporeal membrane oxygenation^23^.

In our study we found an increase of transports due to cardiac, and surgical diseases. Trevisanuto et al.^24^, on the contrary, found a decrease of NETs due to cardiac, and or surgical diseases in Veneto, during the last two decades, probably secondary to better prenatal diagnosis^24–26^.

In our study we found also an increase of transports for neurological diseases, including post-asphyxia neurologic problems. This result may be associated with introduction, in 2009, of therapeutic hypothermia for neonatal asphyxia in few referral centres^27–29^.

The increase of transports of neonates with neurologic diseases may be also explained by the emerging attention to the *neuro-critical care* and the introduction of hyper specialized NICU with better management of neonates with neurologic diseases.

As far as MINT is concerned, in our study we found a reduction of the score during years. The MINT score reflects a first triage of patients, within 72 h of life after birth, to evaluate the severity of the acute clinical condition before activation of NET. The decrease of this parameter reflects a trend of major attention to prenatal diseases and risk at conception. This might be due to an increase of *in-utero* transports, when perinatal conditions might be at risk. Therefore, in primary birth-centres a major attention has been addressed during years to predict eventual perinatal risks and prevent them by a proper transport to tertiary centres with NICU before birth.

The same trend during years was found for the TRIPS. Literature data found that high-risk infants are more at risk for deterioration during transport^16,30^; this includes the smallest and premature infants, neonates requiring delivery room resuscitation, and those with more severe birth defects. The reduction of the TRIPS scores in our study reflect the efficiency of the emergency transport in stabilizing critical ill neonates and the efficiency of this system to facilitate access to tertiary cares centres where these patients might receive intensive therapies. This efficiency reflects a proper system of transports and the ability of the NET team with doctors and nurses specialized, and well equipped for this service, showing a good experience in the management of critical ill neonates. This is also confirmed by an USA study, California, in which the majority of neonatal transports are performed by members of the California Perinatal Transport System (CPeTS), a network of over 100 specialized NICUs who serve to facilitate the transport of critically ill infants to NICUs offering a higher level of care, better able to meet their needs^30^. The high specialization of the transport team allowed to significantly reduce the TRIPS results of these patients, with higher facilities to access tertiary care centres^30^.

## Limitations

The main limit of the present study is related to the retrospective nature of the analysis that usually limits quality and completeness of data.

Another limit of our study was that actual data from patients were tapped from official government database (ISTAT—National Statistic Institute) instead of data from patients who undergone specialised neonatal transport. Therefore, the improvement or worsening in neonatal mortality rates cannot be directly attributed to the set-up of the Neonatal Transport Service.

Moreover, we do not have data on neonatal mortality during the study period, as Sicily lacks of a system of surveillance of perinatal mortality, as that one introduced in Emilia-Romagna. Data on infantile mortality were also available until 2018. We do not have further data updated to 2021.

Finally NETS in Catania has been activated only from 2016, and collected data are not still sufficient to suggest proved evidenced.

## Conclusions

The slight decrease of infantile mortality in Catania during the first three years after the introduction of NET follows the same trend of all Italian territory. This trend reveals a reverse rate for the provinces of Enna, Caltanissetta, and Agrigento, in which the service is not still implemented. These data underline the impact of the NET service in reducing infantile mortality and morbidity.

Nevertheless, these data are still insufficient to suggest a correlation hypothesis, considering the low number of the included patient, and the relative limited time from the introduction of the NET service in Catania.

Our study aimed to show how ultra-specialized skills, such as those required to maintain an appropriate NET service, are fundamental to guarantee the health of vulnerable neonates and to succeed in resuscitation and stabilization of these patients.

Nevertheless, a large sample and data on NET outcomes would clarify the effects of NET implementation on trends in study the rate of infantile mortality and morbidity during over time.

## Data Availability

The datasets generated and/or analysed during the current study are not publicly available due privacy policies but are available from the corresponding author on reasonable request.
